# Two rashes, one patient: Enfortumab vedotin-induced interface dermatitis and pembrolizumab-induced psoriatic disease treated with ixekizumab

**DOI:** 10.1016/j.jdcr.2024.08.030

**Published:** 2024-09-12

**Authors:** Kyle Mueller, Saby George, Paul Bogner, Drew Kuraitis

**Affiliations:** a Jacobs School of Medicine, University of Buffalo, Buffalo, New York; b Department of Dermatology, Roswell Park Comprehensive Cancer Center, Buffalo, New York; c Department of Medicine, Roswell Park Comprehensive Cancer Center, Buffalo, New York; d Department of Pathology, Roswell Park Comprehensive Cancer Center, Buffalo, New York; e Department of Dermatology, Tulane University, New Orleans, Louisiana

**Keywords:** adverse event, cutaneous immune-related adverse event, drug eruption, enfortumab vedotin, immune-related adverse event, immunotherapy, interface dermatitis, ixekizumab, pembrolizumab, psoriasis, psoriatic arthritis

## Introduction

Enfortumab vedotin (EV) and pembrolizumab can be used individually or combined for treatment of locally advanced or metastatic urothelial cancer (UC). EV, an antibody-cytotoxic drug conjugate, causes direct cell toxicity after targeted antibody binding and may induce adverse events (AEs) through unintended cytotoxic effects. Pembrolizumab is an immune checkpoint inhibitor that broadly stimulates T-cell activity, leading to cancer targeting but also risk of immune-mediated AEs in any organ system. Despite their different mechanisms, both agents may induce a variety of cutaneous AEs, with skin reactions occurring in nearly half of patients receiving either drug. Here, we present a patient with high-grade UC who developed 2 distinct cutaneous AEs during treatment with combination EV and pembrolizumab. The first rash was a rapid-onset EV-induced interface dermatitis that resolved with topical corticosteroids, prednisone taper, and discontinuation of EV. The second rash, pembrolizumab-induced psoriasis with psoriatic arthritis, constituted an immune-related AE (irAE), which was successfully managed with ixekizumab.

## Case presentation

**Fig 1:**
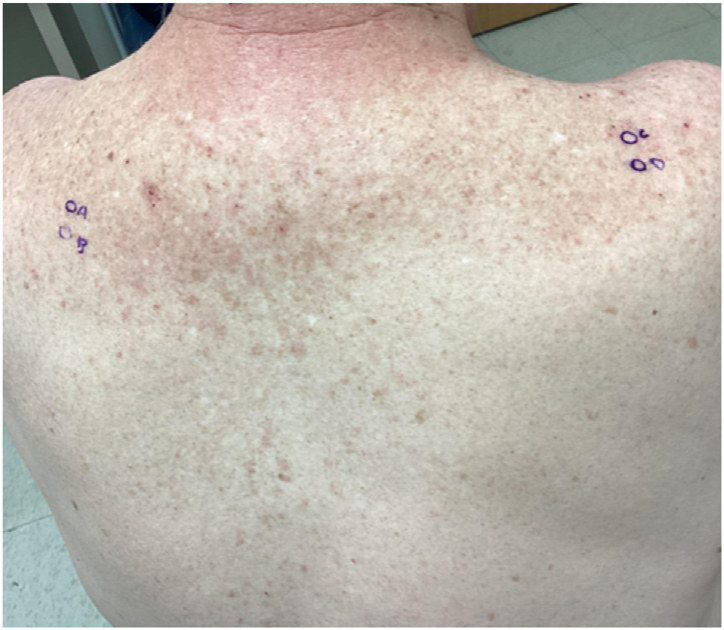
Ill-defined erythematous macules and thin non-scaling papules to the upper back after second treatment with enfortumab vedotin. Biopsy sites marked in blue ink.

**Fig 2:**
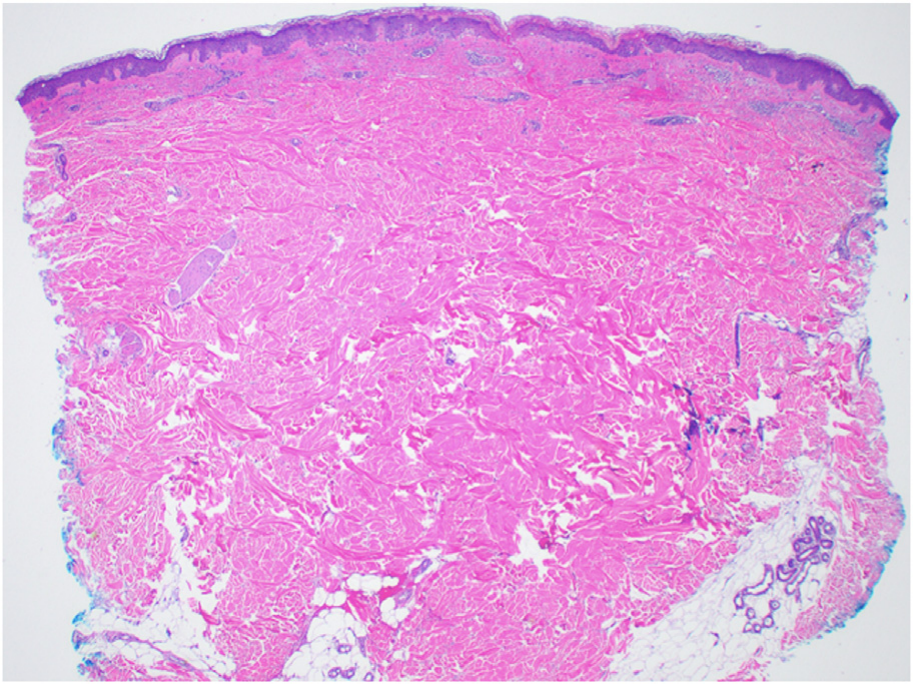
Biopsy of initial cutaneous eruption to the upper back, demonstrating patchy vacuolar interface changes and a superficial perivascular infiltrate. 20×, hematoxylin and eosin.

**Fig 3:**
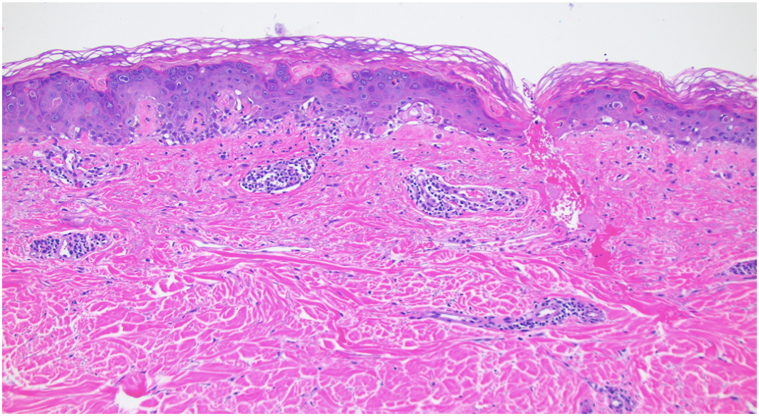
Patchy stretches of vacuolar interface damage and epidermal dyskeratosis with a superficial perivascular infiltrate containing eosinophils. 100×, hematoxylin and eosin.

**Fig 4:**
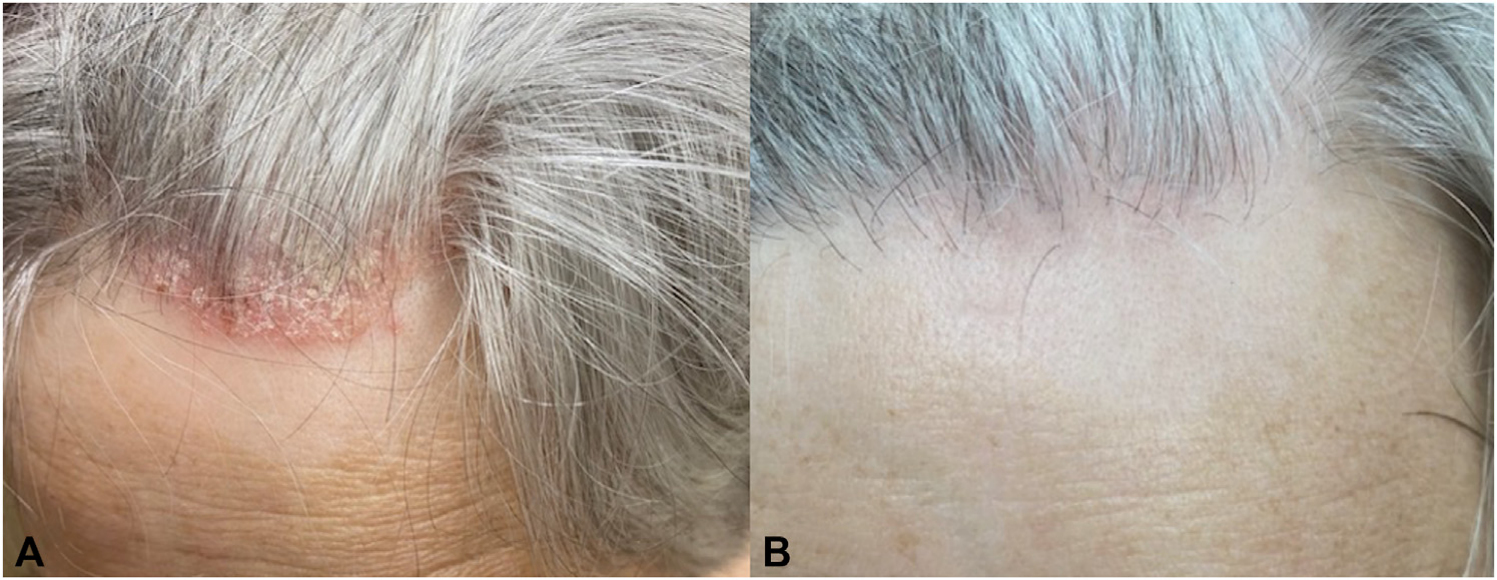
Psoriasiform plaque to the scalp associated with pembrolizumab therapy (**A**) and resolution of plaque with residual post-inflammatory changes after initiation of ixekizumab (**B**).

A 64-year-old man with a history of UC of the bladder, managed with radical cystoprostatectomy 6 years prior, was diagnosed with recurrent UC and received dual-agent EV and pembrolizumab. Four days after infusions, he developed intense itching across his trunk and arms. Suspecting pruritus as a pembrolizumab-related cutaneous irAE, pembrolizumab was held and his next infusion was single-agent EV. His pruritus and quality of life worsened, prompting referral to dermatology clinic. On examination, he had excoriations across the upper trunk and arms, with ill-defined erythematous macules and thin non-scaling papules (Fig 1) approximating 15% body surface area and representing a grade 2 AE; however, pruritus was constant and limiting of daily activities, representing a grade 3 AE. No bullae or vesicles were present. Biopsy demonstrated dyskeratosis and patchy vacuolar interface damage with a perivascular infiltrate containing eosinophils (Figs 2 and 3). He received a prednisone taper, starting at 70 mg, and was also started on triamcinolone 0.1% ointment. He was continued on single-agent pembrolizumab without recurrence of interface dermatitis or pruritus. After cycle 6 of pembrolizumab, he returned to the dermatology clinic for evaluation of an acute-onset rash and joint pains. He had confluent erythematous psoriasiform plaques to the scalp (Fig 4, *A*), accompanied by stiffness in most of his fingers and tenderness to the Achilles tendons, diagnosed as pembrolizumab-induced psoriasis and psoriatic arthritis, representing a grade 2 irAE. He reported a history of scalp psoriasis approximately 20 years prior that had been relatively inactive and untreated. Standard dosing of ixekizumab was initiated with rapid improvement of psoriasis and psoriatic arthritis. He has remained on both ixekizumab and pembrolizumab for over 1 year now without flares of psoriatic disease.

## Discussion

The enfortumab antibody binds nectin-4, highly expressed on bladder tumor cells, and induces cell death via vedotin. Cutaneous AEs of EV are common, possibly due to EV binding to keratinocytes which may also express nectin-4.^1^ Beyond the antibody component, vedotin may cause direct skin toxicity, as other antibody-vedotin conjugates not targeting nectin-4 can cause similar cutaneous reactions.^1^^,^^2^ Cutaneous EV AEs may exhibit patterns of interface dermatitis with or without vacuolization, subepidermal splitting, epidermal spongiosis, dyskeratosis, and varying degrees of mixed inflammatory infiltrates.^1^ These findings commonly appear in drug eruptions and are not specific to EV cutaneous AEs. In more severe cases, subepidermal separation may present as bullous dermatoses, or even mimic toxic epidermal necrolysis, although often without mucosal involvement.

In our patient, his first cutaneous eruption was initially attributed to pembrolizumab. Although pembrolizumab may commonly induce cutaneous eruptions that mimic idiopathic diseases, such as bullous pemphigoid, lichen planus, or psoriasis,^3^ generalized pruritis is often an initial cutaneous irAE and this can present with excoriations and ill-defined erythema. After biopsy, the patient’s interface dermatitis was clinically felt to be secondary to EV instead of pembrolizumab. While histopathologic findings of cutaneous toxicities of either pembrolizumab or EV are not entirely specific, in the authors’ experience, the rapid onset, significant interface damage and dyskeratosis, and presentation relatively quickly after EV administration favored EV as the culprit over pembrolizumab. Worsening of the eruption on EV monotherapy with pembrolizumab discontinuation reinforced EV as the causal agent, and this was further supported with complete resolution of the AE after holding EV indefinitely and continuing pembrolizumab monotherapy. While compound effects of EV and pembrolizumab may have contributed to this AE, elucidation of this process would require further investigation of their interplay. In our case, cessation of EV treatment and support for symptoms managed the reaction. More studies are needed to devise other potential therapies.

After development and resolution of the initial rash, the rapid onset of psoriasis and psoriatic arthritis during continued pembrolizumab therapy represented a second and distinct AE in our patient. Anti-programmed cell death protein (PD-1) immunotherapy may induce psoriatic disease or exacerbate pre-existing psoriatic disease. Induced psoriatic disease may occur weeks to months after treatment initiation.^3^ PD-1 blockade promotes a Th1/Th17 response via enhanced production of interferon γ, interleukin (IL)-2, tumor necrosis factor-α, IL-6, and IL-17 and reduction of Th2 cytokine profiles in patients with cancer.^4^ This pro-inflammatory shift with the overproduction of IL-17 likely contributes to psoriatic disease development. Studies are investigating which patients may develop which cutaneous irAEs, with data suggesting that a patient’s pre-existing immune milieu may drive the development of specific irAEs.^5^ We postulate that irAEs may follow the path of least resistance, manifesting in presentations that the patient previously experienced. Our patient reported a remote and mild history of scalp psoriasis, and thus we favor that his psoriatic irAE is a pembrolizumab-induced recurrence, although more severe than prior reported cutaneous flares, and with *de novo* psoriatic arthritis symptoms.

Psoriatic disease was successfully treated with ixekizumab, an anti-IL-17 humanized monoclonal antibody, one of many interventions reported to treat anti-PD-1 drug toxicity. Ixekizumab has proven clinical effectiveness and safety in the treatment of both psoriasis and psoriatic arthritis and successfully treated psoriasiform irAEs from another immune checkpoint inhibitor, atezolizumab.^6^ Furthermore, prior incidences of pembrolizumab-induced psoriasis resolved with IL-17 blockade^7^ and ixekizumab reportedly resolved a pembrolizumab-induced mixed psoriasiform, lichenoid, and spongiotic eruption.^8^ However, to our knowledge, ixekizumab has not yet been reported as a therapeutic for pembrolizumab-induced psoriasis and psoriatic arthritis.

Skin reactions are common during treatment with either EV or pembrolizumab, around 48% and 42% prevalence, respectively.^9^^,^^10^ Both agents can induce a spectrum of cutaneous AEs, many of which may not be unique or specific to either causal drug. Cutaneous AEs from pembrolizumab may be managed using a variety of steroid-sparing approaches depending on the unique type of irAE that is induced; however, those from EV are often managed with supportive care and dose reduction instead of steroid-sparing agents.^11^ Dermatologists and oncologists alike need to be aware of potential cutaneous AEs and also the potential AEs that may arise from combination therapy. While AEs may be correlated with improved outcomes,^9^^,^^10^ severe reactions may require cessation of life-saving therapies. Roles of the dermatologist include recognizing cutaneous AEs, distinguishing their etiology, providing appropriate therapy to manage AEs, and also to salvage life-saving cancer therapy in the event of severe cutaneous AEs, if the patient is deriving clinical benefit from therapy. This case highlights the possibility of 2 distinct cutaneous AEs in a patient on dual oncologic therapies, expands the spectrum of EV-induced AEs, and underscores ixekizumab as a potential therapeutic for immunotherapy-induced psoriasis and psoriatic arthritis.

## Conflicts of interest

Dr Kuraitis is a consultant for Ortho Dermatologics. Dr George is a consultant or advisor for Bristol Myers Squibb, Bayer, Pfizer, Exelixis, Sanofi, EMD Serono, Seattle Genetics/Astellas, Eisai, Merck, AVEO, QED Therapeutics, Novartis, AstraZeneca. Drs Mueller and Bogner have no conflicts of interest to declare.
